# Does consumption of red grapefruit juice alter naringenin concentrations in milk produced by breastfeeding mothers?

**DOI:** 10.1371/journal.pone.0185954

**Published:** 2017-10-05

**Authors:** Ewa Romaszko, Urszula Marzec-Wróblewska, Anna Badura, Adam Buciński

**Affiliations:** 1 Family Medicine Unit., University of Warmia and Mazury in Olsztyn, Olsztyn, Poland; 2 Nicolaus Copernicus University in Toruń, Collegium Medicum in Bydgoszcz, Faculty of Farmacy, Chair and Department of Biopharmacy, Bydgoszcz, Poland; University of Illinois, UNITED STATES

## Abstract

The content of certain ingredients of human milk, such as flavonoids, depend on the types and amounts of plant products consumed and may vary from woman to woman. The aim of the study was to determine to what extent consumption of an average amount of grapefruit juice (250 ml) affected naringenin content in human milk. A total of 14 breastfeeding mothers were included in the study. The subjects remained on a diet with restricted intake of naringenin for a total of five days except on the third day, when they drank a single serving of 250 ml of grapefruit juice. A considerable subject-to-subject variability in naringenin content was observed in both initial and subsequent determinations. Baseline concentration values, which may reflect naringenin content in the milk produced by the breastfeeding mother who eat an everyday (unmodified) diet, ranged from 420.86 nmol/l to 1568.89 nmol/l, with a mean of 823.24 nmol/l. Switching to the modified diet resulted in a decrease in naringenin concentrations to the mean value of 673.89 nmol/l measured 48 hours after the switch. The highest mean values were observed four and 12 hours after consumption of the juice, equalling 908.25 nmol/l (SD ± 676.84 nmol/l) and 868.96 nmol/l (SD ± 665.54 nmol/l), respectively. Naringenin is commonly found in human milk in quantities expressed in nmol/l, and its concentrations vary from woman to woman. Consumption of 250 ml of red grapefruit juice by breastfeeding mothers does not significantly alter naringenin concentrations in their milk.

## Introduction

Human milk is a species-specific secretion that ensures normal growth and development of the breastfed baby. The composition of human milk changes dynamically throughout lactation according to the changing nutritional needs of the baby [1, 2]. In addition to nutrients, human milk may also contain xenobiotics, including contaminants, medications, and natural non-nutrient substances, such as secondary plant metabolites. The presence of some of them, e.g. flavonoids, depends on the local dietary habits, the types and amounts of plant products consumed, and may vary from woman to woman.

Given the limited number of studies conducted so far and the fact that most of them have focused on isoflavones, it may be reasonably hypothesized that the content of phytochemicals in human milk depends to a certain extent on the breastfeeding woman's diet. Asian women, who commonly consume soy-rich food products, have higher levels of daidzein (approx. 80–100 nmol/l) and genistein (approx. 30–50 nmol/l) during the breastfeeding period compared to Caucasian women, in whom similar levels in breast milk can only be reached after nutritional intervention [3]. In another study, authors confirmed the diet-dependent presence of isoflavones in human milk. They showed that consumption of about 55 mg of these compounds by breastfeeding mothers resulted in an increase in the total quantity of isoflavones from the baseline level of 5.2±2.2 nmol/l (before the intervention) to 70.7±19.2 nmol/l (after the intervention). The decreases in genistein and daidzein concentrations in human milk during the study were not linear [4]. This also applies to quercetin, whose quantity in human milk increases following consumption of foods rich in this compound (e.g. onion) [5]. Naringenin, which is found in citrus fruits, especially pomelo and grapefruit, has been attracting interest among researchers for many years due to its preventive effects in cardiovascular disease, obesity and diabetes mellitus [6–8]. It is also known to affect the bioavailability of certain drugs [9, 10]. Since the discovery of the interaction between grapefruit juice and felodipine, which is caused by the inhibition of cytochrome P450 3A4 by furanocoumarins contained in the juice, it has also been shown that other substances contained in citrus fruits (including naringenin) may affect the bioactivity of certain drugs. Naringenin is a potent inhibitor of organic anion transporting polypeptide (OATP), a compound whose inhibition may impair oral absorption of certain drugs, e.g. fexofenadine [11]. In the light of current knowledge, enantiomers of naringenin should be treated as Pleiotropic, Stereoselective Inhibitors of Cytochrome P450 Isoforms, in other words, as substances that may potentially affect the metabolism of many drugs [12]. Because interactions between different foods and drugs may emerge via mother’s milk, it seems of interest to investigate whether the intake of grapefruit juice typical of normal diet may lead to changes of naringenin concentrations in mother’s milk that would be of pharmacokinetic significance for the sick, breastfed child.

The aim of the study was to determine to what extent consumption of an average amount of grapefruit juice (250 ml) affected naringenin content in human milk.

## Material and methods

### Subjects

The study was registered and approved by the Bioethics Committee of the Warmian-Mazurian Chamber of Medical and Dental Professionals in Olsztyn, Poland as OIL244/14/Bioet. The study participants included a total of 14 breastfeeding women, aged from 25 to 37 years, mean 31.0 (SD ± 3.28) and their 14 healthy, thriving infants aged from 5 to 14 months, mean 7.0 (SD ± 2.94). All the babies were born at term with Apgar scores ranging from eight to ten. Table 1 summarizes the demographic characteristics of the study group.

**Table 1:** Demographic characteristics of the mothers and their infants included in the study [n = 14].

Mothers	mean	SD
Age [years]	31.00	3.28
Weight [kg]	66.64	10.05
Height [cm]	166.43	4.07
BMI [kg/m^2^]	24.02	3.24
**Infants**		
Birth weight [g]	3465.00	516.98
Birth length [cm]	54.93	1.98
Age [months]	7.00	2.94
Weight on the day of the study [g]	8453.57	1107.77

Volunteers for the study were recruited in the period 2014–2015 among the female patients registered with their local family physician’s practice. They were approached directly by their family physician (co-author); 14 mother–child pairs were included in the study–all of them complied with the research protocol, thus, there were no drop-outs. All the volunteers and their infants were in good health and had no history of gastrointestinal disease. None of the subjects were taking any medications.

### Study design

Prior to enrollment, each volunteer was informed about the course and aim of the study, and signed the informed consent. They were also provided with oral and written instructions on the diet that disallowed consumption of any naringenin-containing foods and beverages. The volunteers agreed to follow the diet and not to take any vitamin supplements or any other food supplements for the duration of their participation in the study. They followed the diet for a total of 5 days, refraining from eating foods containing larger quantities of naringenin, such as citrus fruits, citrus fruit juices, citrus fruit pulps, tomatoes and tomato-containing food products, aromatic herbs. For the first two days of the study (pre-exposure) the volunteers collected breast milk samples every 12 hours, each in the amount of four to six ml. On the third day of the study they drank a single 250 ml serving of red grapefruit juice (a 250 ml bottle) in the morning on an empty stomach. In this part of the experiment, the volunteers collected breast milk samples at the following time points: 0 (directly before drinking the juice) and 1, 2, 3, 4, 5, 6, 12, 18, 24, 36 hours post-exposure. Milk samples were collected from full breasts. Milk was collected into chemically pure disposable plastic test tubes. In order to avoid trace contamination, the mothers did not use breast pumps but collected the milk by hand expression.

The samples were frozen by the volunteers at –20°C, regularly collected from the volunteers by the investigator, transported in a laboratory deep freezer, and frozen to –80°C until analysis. Each sample was labelled with the volunteer's initials and with the date and time of sampling. During the study, we remained in constant telephone contact with the volunteers. A commercial 100% grapefruit juice originating from ecological farms in Israel, in 250 ml bottles, were used in the study. The juice was purchased from a local grocery wholesaler. All the bottles were labelled with the same lot number.

### Chemical analysis

#### Chemicals and standards

β-glucuronidase (G0751), ethyl acetate, hexane, isopropyl alcohol, methanol, Na_2_-ethylenediaminetetraacetic acid (E5134) and pepsin (P7000) were purchased from Sigma-Aldrich Co. (St. Louis, MO, USA). L-Ascorbic acid and NaOH were obtained from Avantor Performance Materials Poland S.A. (Gliwice, Poland). HCl and formic acid were purchased from Merck KGaA (Darmstadt, Germany). Naringenin (N5893) was obtained from Sigma-Aldrich Co. (St. Louis, MO, USA). Millipore Milli-Q system (Millipore Co., Millford, MA, USA) was used to produce ultrapure water.

#### Naringenin extraction

Naringenin was extracted according to the method described by Song et al. with minor changes [13]. In brief, an aliquot (0.5 ml) of undiluted human milk was defatted two times with 2-ml of hexane. After the lipid removal, 25 μl of L-Ascorbic acid (2.7 mmol/l in ultrapure water), 25 μl of Na_2_-ethylenediaminetetraacetic acid (2.2 mmol/l in ultrapure water) and 3 ml of pepsin (40 mg/ml in 0.1 N HCl) were added. The samples were incubated in a ThermoMixer C (Eppendorf AG., Germany) for 15 min at 37°C and at 300 rpm. Then, the pH of the sample was adjusted to 4.5 with 1.0 N NaOH and 3.86 kU of β-glucuronidase with sulfatase contaminant was added. Afterwards the samples were incubated for 45 min at 37°C and at 300 rpm in a ThermoMixer C. After deconjugation the potential metabolites (glucuronides and sulfate derivatives) to aglycones using β-glucuronidase/sulfatase treatment, naringenin was extracted with a 3-ml aliquot of ethyl acetate of tree times, dried under vacuum and resuspended in 200 μl of mobile phase B before analysis.

The same extraction procedure was applied for the samples of grapefruit juice.

#### HPLC apparatus and analysis

Naringenin analysis was done on a Shimadzu HPLC system (Kyoto, Japan), equipped with binary pump (LC-20 AD), degasser (DGU-20A3), refrigerated autosampler (SIL-20 AC, set at 8°C), a column oven (CTO-20 AC, 30°C), a diode array detector (SPD-M20A) and a mass spectrometry detector (2010EV). Separations were performed on Kinetex XB-C18 column (100×2.1 mm, 2.6 μm particle size, Phenomenex) at 0.2 ml min^−1^ flow rate. The mobile phase consisted of A: 0.4% formic acid in ultrapure water and B: 0.4% formic acid, 0.4% isopropyl alcohol in methanol. The initial conditions of gradient program were set at 98:2 (A/B) followed by a linear gradient to 55:45 (A/B) at 15 min, 20:80 (A/B) at 30 min, 98:2 (A/B) at 35 min and at 40 min, stop. The injection volume was 10 μl. Naringenin was quantified at 287 nm. MS spectra were acquired in positive ion mode over the range of m/z 50–500, both using SCAN and SIM acquisition mode (the set of SIM included 273,25 m/z). The following ESI parameters were applied—1.5 kV interface voltage, 1.5 kV detector voltage, desolvation at 230°C, drying gas flow at 15.0 l min^−1^, nebulizing gas flow at 1.5 l min^−1^. The data were processed with LC-MS solution 3.40 software (Kyoto, Japan). Quantitation of naringenin was accomplished using calibration curves created from serial dilutions of naringenin standard stock solutions. R^2^ value was 0.999. The LOD (limit of detection) and LOQ (limit of quantitation) were found to be 367.29 nmol/l and 1469.18 nmol/l, respectively.

### Calculations and statistics

Statistical analyses were performed with STATISTICA version 12 software (StatSoft, Inc., www.statsoft.com). The mean value, standard deviation and median were calculated for all analyzed parameters. The Shapiro-Wilk test was employed to detect all departures from normality. Levene's test was used to assess the equality of variances for variables calculated in different groups. When the assumptions referring to normality and equality of variances were met, t-Student test was employed; otherwise the Mann–Whitney U test was used. To compare repeated measures we employed Friedman test. We adopted a statistical significance level of p = 0.05. To calculate the terminal phase rate constant and the area under the curve (AUC) Kinetica 5.0 software was used (Thermo Scientific™, www.thermofisher.com).

## Results

### Analysis of the grapefruit juice

Naringenin and naringin were identified in the test material. Naringenin and naringin were present at concentrations of 13969.62 nmol/l (SD ± 555.93) and 93601.19 nmol/l (SD ± 1922.33), respectively.

### Analysis of the human milk

**Fig 1:**
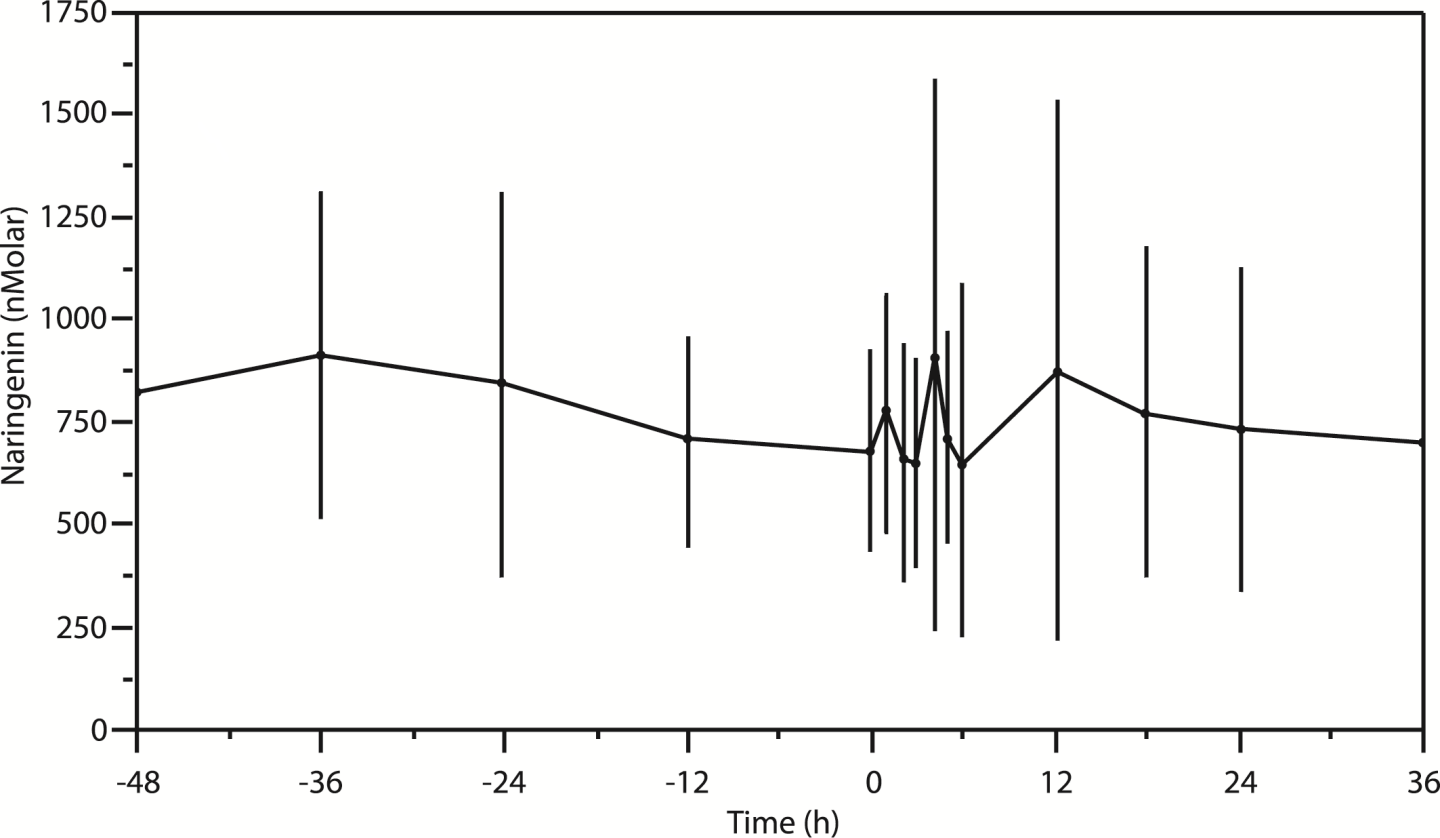
Mean naringenin concentrations in human milk. Friedman test (N = 10, df = 14) = 18.903 p = 0.169.

A considerable subject-to-subject variability in naringenin content was observed in both initial and subsequent determinations. Naringenin concentrations in the initial samples collected from the subjects before the initiation of the naringenin-restricted diet ranged from 420.86 nmol/l to 1568.89 nmol/l, averaging 823.24 nmol/l (SD ± 284.63 nmol/l), and may be treated as a measure reflecting naringenin content in human milk during everyday (unmodified) diet. Switching to the naringenin-restricted diet resulted in a decrease in naringenin concentrations to the mean value of 673.89 nmol/l (SD ± 247.64 nmol/l) measured 48 hours after the switch (the zero hour, see Fig 1). Grapefruit juice consumption resulted in an increase in naringenin content in the volunteers' milk at one, four and 12 hours post-exposure (Fig 1).

The highest values were observed at 4 and 12 hours post-exposure and averaged 908.25 nmol/l (SD ± 676.84 nmol/l) and 868.96 nmol/l (SD ± 665.54 nmol/l), respectively. A decrease in the mean concentration to 701.59 nmol/l (SD ± 370.82 nmol/l) was observed at 36 hours post-exposure (again, while the subjects were on the naringenin-restricted diet). Fig 1 depicts mean naringenin concentrations in human milk. The pharmacokinetic parameters for the pre-exposure segment and for the post-exposure segment are shown in Table 2.

**Table 2:** Pharmacokinetic parameters.

Parameter	Pre-exposure Mean; SD; median; n	Post-exposure Mean; SD; median; n	P value
**C_max_ [nmol/l]**	910.1; 407.1; 762.2; 14	908.3; 676.8; 713.8; 14	0.221
AUC [(nmol/l)*h]	27455; 7218; 26515; 14	27419; 13473; 23715; 14	0.594
**K_el_ [h^-1^]**	0.020; 0.010; 0.020; 5	0.014; 0.009; 0.013; 11	0.304

Pre-exposure and post-exposure AUC values were calculated for -36 h to 0 h and 0 h to 36 h, respectively. Trapezoidal method was used to compute AUC.

The elimination constant (K_el_) was calculated for last points for the pre-exposure segment and for the post-exposure segment.

## Discussion

### Flavones in human milk

Citrus fruits, which are a rich source of flavanones, are widely consumed in many countries of the world [14, 15]. Flavanones represent a small group of compounds, including glycosides of naringenin, mainly naringin (4’, 5, 7-trihydroxyflavanone-7-rhamnoglucoside), which is the predominant flavanone in grapefruit. Naringenin is also present, although in much smaller quantities, in other other citrus fruits and in tomatoes [16, 17]. A limited number of studies have been conducted to confirm the presence of flavonoids in human milk. Song et al., evaluated the contents of various flavonoids on the 1st, 4th and 13th day of lactation, identifying a wide range of naringenin concentrations from 64.1 nmol/l to 722.0 nmol/l in individual volunteers [13]. The study did not assess changes in the concentrations of these compounds after modification of the diet, but only the presence of these compounds. According to the authors of the above paper, the differences in concentrations could have been a result of individual nutritional preferences of the breastfeeding women.

### Naringenin absorption and content in human milk

In our study, naringenin concentrations in the initial, baseline milk samples, i.e. before the switch from the everyday diet, were slightly higher and were characterized by a wide range of values from 420.86 nmol/l to 1568.89 nmol/l, averaging 823.24 nmol/l (SD ± 284.63 nmol/l). Switching to the naringenin-restricted diet by the volunteers resulted in a decrease of naringenin content of their milk to the mean value of 673.89 nmol/l (SD ± 247.64), while consumption of grapefruit juice caused an increase at one, four and 12 hours post-exposure with the peak mean concentration of 908.25 nmol/l (SD ± 676.84) at four hours post-exposure. The rate of absorption of flavanones from the gastrointestinal tract is determined by the form in which they have been administered. Oral administration of the aglycon leads to its rapid absorption [18]. Blood concentration begins to rise within 20 minutes and peaks between 3.5 and approximately 5 hours after exposure [19, 20]. Glycosides, on the other hand, show a slower absorption. Their absorption requires prior hydrolysis, which occurs in the large intestine and is catalyzed by the enzymes of the intestinal microflora [21]. As a result, glycosidic bonds are cleaved and aglycons are released from the saccharide compounds. The released aglycons may be absorbed by the colonic mucosa or further metabolized by the bacterial microflora with the formation of low-molecular-weight phenolic compounds, such as phenylacetic and phenylpropionic acids and their derivatives.

In our study, we observed increases in naringenin concentration at three time points: first, within the first hour post-exposure, second, at four hours post-exposure and third at 12 hours post-exposure (Fig 1).

Friedman’s ANOVA (N = 10, df = 14) = 18,903; (p = 0.169) did not demonstrate significant differences between naringenin concentrations in the milk samples, leading to a conclusion that consumption of a glass of grapefruit juice did not affect naringenin levels in milk to a significant extent. Also the post-exposure concentration values did not differ significantly from the values observed with normal diets consumed by the breastfeeding mothers. No significant differences were also shown for AUC values before the intake of the juice (-36 to 0 h) and after the intake of the juice (0 to 36 h) and for the mean values of C_max_ and K_el_. The results of these analyses are provided in Table 2.

The concentration fluctuations suggest a rapid absorption of the aglycon, delayed absorption of the glycoside and the likely significant role of the enterohepatic circulation in the metabolism of flavanones. In grapefruits, naringenin is mainly found in the form of glycosides [17]. The analysis of the juice used in our study has, however, shown the presence of both the glycoside (naringin) and the aglycon (naringenin). Technological processes employed in the food industry during the manufacture of grapefruit juice may alter the content and structure of naringenin compounds.

In their study on the animal model (the rat), Felgines et al. analysed the kinetics of serum naringenin concentrations after feeding the animals with the aglycon, 7-glucoside and 7-rhamnoglucoside [22]. Increases in naringenin concentrations were observed at three hours of the experiment after using the aglycon and 7-glucoside, and at ten hours after using 7-rhamnoglucoside. At 24 hours post-exposure, in each of the analzsed groups, high concentrations of naringenin were still present.

In our results, after 48 hours of the diet pre-exposure and after 36 hours of the diet post-exposure, we also observed high concentrations of naringenin, averaging 673.89 (SD ± 247.64) and 701.59 (SD ± 370.82) nmol/l, respectively. According to limited literature data, certain phenolic compounds could accumulate in the breast gland. This applies to isoflavones (daidzein, genistein), chalcones (xanthohumol) and flavonones (isoxanthohumol) [23, 24]. There have been no reports so far to confirm naringenin deposition in the breast gland. This mechanism cannot, however, be ruled out. The biphasic increase of isoflavone concentrations in human milk has been reported in breastfeeding women after consumption of soy, with peak concentrations being achieved 10–14 hours post-exposure [4]. Based on the mechanisms of biosynthesis of human milk, low-molecular-weight substances (below 150 Da) should readily diffuse into human milk, while the diffusion of those whose molecular weight exceeds 500 Da should be more difficult [25]. The molecular weight of naringenin is somewhere between (272.26 g/mol). Assuming that no active transport mechanism exists for naringenin excretion into human milk, the concentration of this compound in milk should be proportional to its serum concentration (diffusion). It should, however, be borne in mind that the biosynthesis of human milk occurs both between and during the feeds. It is also common knowledge that babies not always and not completely empty the breast, and that they may not content themselves with just one but empty both breasts [26]. These factors may affect the differences in concentrations, diffusion rates and, as a consequence, the subject-to-subject variability in naringenin concentrations in human milk.

### Kinetic and limitations

Research concerning the content of breastfeeding mother’s milk always runs the risk of being flawed by physiological changes of milk content. A child breastfed at request may empty only one breast, or both breasts or none of them. This changes the volume of the remaining substances after a feed and may be the reason for large differences in the obtained results. This refers to all substances diffusing to milk. However, we aimed at detecting the range of naringenin concentrations after exposition do a typical dietary dose in the context of potential interactions between foods and drugs. The realization of this aim must account for ethical conditions or be limited to data consistent with everyday situations. In order to minimize the potential flaw, milk was collected before a feed from a full breast. In our experiment, the diet was established on the basis of the literature, taking into account the fact that the highest flavanone content is found primarily in citrus fruits and selected vegetables [27]. We cannot rule out that some of the volunteers inadvertently violated the dietary regime by consuming a naringenin-containing food product that was not on the list we provided. The calculated values of the elimination constant pre- and post-exposure were very similar (0.020 and 0.013, respectively, and did not differ significantly; see Table 2), which indirectly suggests that this, however, was not the case. Additionally, pre- and post-exposure decreasing of the naringenin concentration suggest that in both time segment of the naringenin elimination phase was observed. Moreover, similar values of others pharmacokinetic parameters calculated in this study indicated that the concentration of naringenin in a single 250 ml serving of red grapefruit juice used in the experiment corresponds to naturally occurring naringenin concentration in the normal diet of breastfeeding women. Maximum concentration (C_max_) of naringenin for pre-exposure interval was 910.10 nmol/l, when for post-exposure segment was 908.25 nmol/l.

### Potential biological significance

Our study evaluated the presence and the kinetics of naringenin concentrations in human milk. It did not provide an answer about the biological significance of this compound in lactation. Flavonoids commonly consumed with food are xenobiotics. They may beneficially affect the human body. As antioxidants, they may increase the anti-oxidative potential of human milk, although their toxicity to humans, and to infants in particular, cannot be ruled out. We also cannot exclude the fact that this could be just one of many routes of elimination for flavonoid compounds from the body of the mother, as is the case with dioxin [28].

## Conclusions

Naringenin is commonly found in human milk in quantities expressed in nmol/l, and its concentrations vary from woman to woman. Consumption of 250 ml of red grapefruit juice by breastfeeding mothers does not significantly alter naringenin concentrations in their milk.

## Supporting information

S1 Data Aggregate dataset. (XLSX)
